# Effects of glucose oxidase on growth performance, clinical symptoms, serum parameters, and intestinal health in piglets challenged by enterotoxigenic *Escherichia coli*

**DOI:** 10.3389/fmicb.2022.994151

**Published:** 2022-10-03

**Authors:** Weiwei Wang, Ruiyan Xie, Qingyun Cao, Hui Ye, Changming Zhang, Zemin Dong, Dingyuan Feng, Jianjun Zuo

**Affiliations:** Guangdong Provincial Key Laboratory of Animal Nutrition Control, College of Animal Science, South China Agricultural University, Guangzhou, China

**Keywords:** antibiotic alternative, bacterial infection, weaned piglet, body weight gain, immune status, intestinal disruption, gut microbiota

## Abstract

Glucose oxidase (GOD) could benefit intestinal health and growth performance in animals. However, it is unknown whether GOD can protect piglets against bacterial challenge. This study aimed to evaluate the protective effects of GOD on growth performance, clinical symptoms, serum parameters, and intestinal health in piglets challenged by enterotoxigenic *Escherichia coli* (ETEC). A total of 44 male weaned piglets around 38 days old were divided into four groups (11 replicates/group): negative control (NC), positive control (PC), CS group (PC piglets +40 g/t colistin sulfate), and GOD group (PC piglets +200 g/t GOD). All piglets except those in NC were challenged with ETEC (*E. coli* K88) on the 11th day of the experiment. Parameter analysis was performed on the 21st day of the experiment. The results showed that the ETEC challenge elevated (*p* < 0.05) the rectal temperature and fecal score of piglets at certain time-points post-challenge, reduced (*p* < 0.05) serum glucose and IgG levels but increased (*p* < 0.05) serum alanine aminotransferase activity, as well as caused (*p* < 0.05) intestinal morphology impairment and inflammation. Supplemental GOD could replace CS to reverse (*p* < 0.05) the above changes and tended to increase (*p* = 0.099) average daily gain during the ETEC challenge. Besides, GOD addition reversed ETEC-induced losses (*p* < 0.05) in several beneficial bacteria (e.g., *Lactobacillus salivarius*) along with increases (*p* < 0.05) in certain harmful bacteria (e.g., *Enterobacteriaceae* and *Escherichia*/*Shigella*). Functional prediction of gut microbiota revealed that ETEC-induced upregulations (*p* < 0.05) of certain pathogenicity-related pathways (e.g., bacterial invasion of epithelial cells and shigellosis) were blocked by GOD addition, which also normalized the observed downregulations (*p* < 0.05) of bacterial pathways related to the metabolism of sugars, functional amino acids, nucleobases, and bile acids in challenged piglets. Collectively, GOD could be used as a potential antibiotic alternative to improve growth and serum parameters, as well as attenuate clinical symptoms and intestinal disruption in ETEC-challenged piglets, which could be associated with its ability to mitigate gut microbiota dysbiosis. Our findings provided evidence for the usage of GOD as an approach to restrict ETEC infection in pigs.

## Introduction

Enterotoxigenic *Escherichia coli* (ETEC) is one of the most prevalent pathogenic bacteria associated with various health disorders in farm animals because it has a strong capacity to colonize on intestinal epithelia by binding to the specific receptors in the brush border membrane, followed by secretion of multiple enterotoxins (Fleckenstein et al., 2010). For piglets, ETEC infection brings about huge economic losses due to the induction of postweaning intestinal disruption, causing impaired growth performance along with elevated morbidity and mortality (Laird et al., 2021; Pupa et al., 2022). Although antibiotics were widely employed to control *E. coli* infection in animals during the past few decades, a stipulation had been drawn for the inhibition of antibiotics used in animal diets in many countries and areas considering the increasing of resistant bacteria and antibiotic residues. Thereby, there is a high demand to explore the available approaches to limit the detriments of ETEC challenge in piglets. Recent studies have focused on the importance of glucose oxidase (GOD), a kind of feed enzyme, as a potential substituent for antibiotics in animals (Wu et al., 2019, 2020).

Glucose oxidase is an aerobic dehydrogenase derived from the fermentation of certain fungal strains such as *Aspergillus* and *Penicillium* (Wu et al., 2020). This enzyme specifically oxidizes β-D-glucose into gluconic acid and simultaneously generates hydrogen peroxide following the consumption of a mass of oxygen (Wong et al., 2008). It is known that GOD has gained much attention due to its benefits on the growth and health of animals (Wu et al., 2019, 2020), which might be associated with the following mechanisms: (1) GOD-induced consumption of oxygen assists with forming anaerobic microenvironment in the gut, which can enhance intestinal antioxidant action by inactivating free radicals and can promote intestinal microecological balance by favoring the growth of beneficial bacteria in the gut (Wang et al., 2018; Wu et al., 2019). (2) The produced gluconic acid under GOD catalysis may inhibit pathogens in the gut and enhance digestive enzymes activities by functioning as an acidifier (Wu et al., 2019; Zabek et al., 2020), besides, gluconic acid can transform into butyric acid in the hindgut under microbial fermentation to act as a critical nutritional component for intestinal epithelial cells, thus supporting cell renewal and repair as well as anti-inflammation of the intestinal mucosa (Tsukahara et al., 2002; Biagi et al., 2006). (3) Generation of hydrogen peroxide catalyzed by GOD is favorable for defending against bacterial invasion due to its broad-spectrum antibacterial effect (Murphy and Friedman, 2019). Previously, several studies have validated the positive effects of GOD addition on growth performance and gut health in chickens (Wu et al., 2019, 2020; Meng et al., 2021) and pigs (Tang et al., 2016; Dang et al., 2021; Sureshkumar et al., 2021) under the non-challenge condition. Moreover, GOD might be profitable for guarding against bacterial infection, as supported by the study of Zhao et al. (2022) who observed that GOD addition attenuated *Clostridium perfringens*-induced necrotic enteritis of broilers by improving intestinal structure and functions. However, it is unknown whether GOD can protect pigs against bacterial infection. Based on the unique characteristics of GOD catalyzed reaction, we assumed that GOD could alleviate ETEC-induced detriments to piglets. This study was thus conducted to investigate the putatively protective effects of dietary GOD addition on growth performance, clinical symptoms, serum parameters, and intestinal health in piglets challenged by ETEC.

## Materials and methods

### Animals and experimental design

The experimental animal protocols for this study were approved by the Animal Care and Use Committee of South China Agricultural University. Duroc × (Landrace × Yorkshire) crossbred weaned piglets (male) around 33-day old with the same litter origin were individually reared in steel pens in an environmentally controlled room, maintained at approximately 25°C. Each pen had one single space shelf feeder and one bowl drinker. After acclimation to the environment and basal diet for 5 days, a total of 44 male piglets were selected based on their individual weight and divided into four treatment groups with 11 replicates per group (one piglet per replicate). The initial body weight (BW) of piglets was similar across groups. The treatment groups were as follows: negative control (NC, received a basal diet with no challenge), positive control (PC, received a basal diet with the ETEC challenge), CS group (PC piglets supplemented with 40 g/t colistin sulfate, a typical antibiotic against Gram-negative bacteria), and GOD group (PC piglets supplemented with 200 g/t GOD). The commercial GOD preparation (10,000 U/g, Qactive Bio-Sciences Co., Ltd., Kunming, China) was produced from *Aspergillus niger* fermentation. According to the manufacturer’s information, this supplement can withstand the severe environment within the digestive tract due to high resistance to gastric acid, cholate, and protease. The dosage (200 g/t) of GOD was selected based on several preliminary experiments in our laboratory. The composition and nutrient levels of the basal diet are displayed in Supplementary Table 1. This experiment lasted for 21 days, during which piglets had free access to the drinking water and powdery feed. Pigs were exposed to a combination of natural and artificial light for 16 h/day.

### Oral challenge and sampling

The *E. coli* K88 strain (CVCC225, China Veterinary Culture Collection Center, Beijing, China), a typical ETEC prevalent in pig production, was inoculated in lactose broth and cultured in an incubator shaker (37°C, 180 r/min) overnight. The bacteria were enumerated by plating on MacConkey agar at 37°C for 24 h. On the 11th day of the experiment, each piglet in PC, CS, and GOD groups was orally gavaged with 5 ml of *E. coli* K88 culture (5 × 10^9^ CFU/ml), while NC piglets received the same amount of lactose broth. On the 21st day of the experiment, blood was taken from the precaval vein of all piglets (11 piglets per group) and serum samples were then obtained by centrifugation of blood at 3,000 rpm for 10 min at 4°C. Piglets were then sacrificed for the separation of the gastrointestine. Afterward, the midpoints of the duodenum, jejunum, and ileum of each piglet were harvested and separated into two segments, one of which was fixed in 10% formalin, and the other one was frozen in liquid nitrogen and preserved at −80°C. Besides, ileal digesta was collected from each piglet for gut microbiota analysis.

### Measurements of growth performance and clinical symptoms

The BW of piglets was weighed on 1, 10, and 21 days of the experiment for calculating the average daily gain (ADG) from 1 to 10 days (pre-challenge period), 11 to 21 days (challenge period), and 1 to 21 days (overall period) of the experiment. At 0 (baseline), 9, 24, 48, 120, and 192 h after challenge (10 days of the experiment), clinical symptoms including the rectal temperature and fecal score of piglets were determined using an electronic thermometer and a three-grade scoring system (Marquardt et al., 1999), respectively.

### Assay of serum parameters

Serum total protein (TP), albumin, and glucose were quantified using biuret colorimetry, bromocresol green colorimetry, and glucose oxidase method, respectively. Serum alanine aminotransferase (ALT) activity was determined using the microplate method. Serum immunoglobulins (Ig), including IgG, IgA, and IgM levels, were measured by the double antibody sandwich ELISA. The commercial kits applied for the determination of the above parameters were purchased from Jiancheng Bioengineering Institute (Nanjing, China).

### Determination of gastrointestinal pH and intestinal morphology

A DELTA320 pH meter (Mettler Toledo, Switzerland) was used to determine the pH values of digesta at three different locations within each stomach, duodenum, jejunum, ileum, cecum, colon, and rectum. The average of three measurements represented the final pH of each gastrointestinal segment.

The duodenal, jejunal, and ileal tissues fixed in formalin were embedded in paraffin and stained by hematoxylin–eosin to obtain cross-sections. Ten representative and intact villi from each section were selected for morphology measurement using a light microscope equipped with the Leica Qwin image analysis system. Villus height (VH) was measured from the villous tip to the villus-crypt joint, while crypt depth (CD) was viewed as the depth of invagination between adjacent villi. Afterward, the ratio of VH to CD (VCR) was calculated.

### RNA isolation and real-time PCR

Total RNA of duodenum and ileum was isolated and purified using the FastPure Cell/Tissue Total RNA Isolation Kit V2 (Vazyme Biotech. Co. Ltd., Nanjing, China) according to the manufacturer’s instructions. The isolated RNA was dissolved in RNase-free water and quantified with a NanoDrop-2000 spectrophotometer (Thermo Fisher Scientific, Waltham, United States). RNA purity was estimated by examining the ratio of absorbance at 260–280 nm. RNA integrity was checked by detecting the 18 and 28S bands after electrophoresis in 1% agarose gels. Thereafter, RNA was reverse transcribed to cDNA samples using the HiScript II qRT SuperMix for qPCR (Vazyme Biotech. Co. Ltd., Nanjing, China). Real-time PCR for assaying gene expression was implemented using the 2 × ChamQ Universal SYBR qPCR Master Mix (Vazyme Biotech. Co. Ltd., Nanjing, China) in a CFX96Touch RT-PCR system (Bio-Rad Laboratories, Hercules, United States). Primer information for the reference gene (reduced glyceraldehyde-phosphate dehydrogenase, GAPDH) and target genes including interleukin (IL)-6, IL-8, and tumor necrosis factor (TNF)-α are shown in Table 1. The relative mRNA expression of target genes was calculated using the 2^-ΔΔCt^ method (Livak and Schmittgen, 2001).

**Table 1:** Primers used for RT-PCR.

Genes^1^	Primer sequences (5′-3′)	Product size (bp)
GAPDH	F: GTGAAGGTCGGAGTGAACGGATTT	253
	R: CCCATTTGATGTTGGCGGGAT	
IL-6	F: GCTGCAGTCACAGAACGAGT	167
	R: GGACAGGTTTCTGACCAGAGG	
IL-8	F: TGAGAAGCAACAACAACAGCA	129
	R: CAGCACAGGAATGAGGCATA	
TNF-α	F: GCATCGCCGTCTCCTACCA	204
	R: CCTGCCCAGATTCAGCAAAGT	

1 GAPDH, reduced glyceraldehyde-phosphate dehydrogenase; IL, interleukin; TNF, tumor necrosis factor.

### High-throughput sequencing of gut microbiota

Bacterial DNA was extracted from ileal content (eight samples were randomly selected from each group) using a NucleoSpin® DNA Stool kit (MACHEREY-NAGEL company, Germany). The quality and concentration of extracted DNA were validated with gel electrophoresis and Nanodrop 2000 (Thermo Fisher Scientific, Waltham, United States). Bacterial 16S rDNA sequences spanning the variable regions V3–V4 were amplified using primers 338 F (5′- ACTCCTACGGGAGGCAGCAG −3′) and 806 R (5′- GGACTACHVGGGTWTCTAAT −3′). The PCR products were sequenced by the Allwegene BioTech. Inc. (Beijing, China) on an Illumina Novaseq platform (Illumina, San Diego, United States) Miseq PE300 platform. According to a 97% sequence similarity, the effective reads were clustered into operational taxonomic units and classified at different taxonomic levels. Bacterial α-diversity was analyzed using the MOTHUR program. Bacterial β-diversity was evaluated by the partial least squares discriminant analysis (PLS-DA). Functional contents of gut microbiota were predicted using the Phylogenetic Investigation of Communities by Reconstruction of Unobserved State (PICRUSt).

### Statistical analysis

Data are expressed as mean ± SD. All data except those of gut microbiota were analyzed by one-way ANOVA in the general linear model procedure of SPSS 20.0. Differences among treatments were detected by Duncan′s multiple comparisons. Kruskal-Wallis tests were used to detect differences in the abundances of bacterial members and predicted pathways among groups. Significance defined as *p* < 0.05 and 0.05 < *p* < 0.10 was considered as a tendency toward significance.

## Results

### Growth performance

There were no differences (*p* > 0.05) in the BW on days 1, 10, or 21 along with ADG during days 1–10 (pre-challenge period), days 11–21 (challenge period), or days 1–21 (overall period) among groups (Table 2). However, the BW of piglets on day 21 coupled with ADG during the challenge period and overall period were numerously lower in the PC group than in the NC group. In comparison, the GOD group tended (*p* = 0.099) to have a higher ADG during the challenge period compared with the PC group.

**Table 2:** Effect of dietary treatments on growth performance^1^ in piglets challenged by enterotoxigenic *Escherichia coli* (ETEC).

Treatments^2^	BW (kg)			ADG (g)		
	1 day	10 days	21 days	1–10 days	11–21 days	1–21 days
NC	10.57 ± 1.33	12.72 ± 1.20	15.54 ± 1.42	220.67 ± 27.75	263.25 ± 34.02	253.33 ± 37.50
PC	10.47 ± 1.13	12.75 ± 1.40	14.73 ± 1.29	221.20 ± 40.55	236.20 ± 21.09	231.30 ± 14.97
CS	10.56 ± 1.41	13.60 ± 1.36	16.40 ± 1.16	241.50 ± 16.09	267.50 ± 30.74	265.79 ± 19.89
GOD	10.56 ± 1.12	12.75 ± 0.78	16.15 ± 0.92	223.20 ± 32.94	281.25 ± 7.59	266.35 ± 27.40
*p* value	0.998	0.522	0.199	0.761	0.099	0.210

1 BW, body weight; ADG, average daily gain.

2 NC, negative control (piglets were free of challenge); PC, positive control (piglets were challenged with ETEC on the 11th day of the experiment); CS, PC piglets supplemented with 40 g/t colistin sulfate; and GOD, PC piglets supplemented with 200 g/t glucose oxidase.

### Clinical symptoms

The rectal temperature of piglets before the challenge was not different (*p* > 0.05) among groups (Table 3). It was noticeable that PC piglets displayed an increase (*p* < 0.05) in the rectal temperature at 9, 24, and 192 h post-challenge compared with NC piglets. Besides, the rectal temperature at 9, 24, 48, and 192 h post-challenge was decreased (*p* < 0.05) in the CS group vs. the PC group, while the reduction (*p* < 0.05) in the rectal temperature of the GOD group relative to PC group occurred only at 9 and 192 h post-challenge. The fecal score at 9 and 48 h post-challenge in both CS and GOD groups was lower (*p* < 0.05) than that in the PC group but did not differ (*p* > 0.05) from the NC group (Table 4).

**Table 3:** Effect of dietary treatments on the rectal temperature in piglets post enterotoxigenic *Escherichia coli* (ETEC) challenge.

Treatments^1^	0 h	9 h	24 h	48 h	120 h	192 h
NC	38.89 ± 0.34	39.18 ± 0.30^b^	39.27 ± 0.23^b^	39.50 ± 0.23^a^	39.58 ± 0.36	38.92 ± 0.20^b^
PC	39.06 ± 0.47	39.78 ± 0.4^a^	39.84 ± 0.41^a^	39.84 ± 0.47^a^	39.88 ± 0.43	39.60 ± 0.34^a^
CS	38.80 ± 0.41	38.95 ± 0.22^b^	39.36 ± 0.29^b^	39.07 ± 0.37^b^	39.73 ± 0.33	39.03 ± 0.32^b^
GOD	38.97 ± 0.37	39.33 ± 0.54^b^	39.62 ± 0.43^a^ ^b^	39.62 ± 0.45^a^	39.69 ± 0.27	38.80 ± 0.33^b^
*p* value	0.619	0.001	0.013	0.003	0.428	<0.001

a,b Values with different superscripts within the same column differ significantly (*p* < 0.05).

1 NC, negative control (piglets were free of challenge); PC, positive control (piglets were challenged with ETEC on the 11th day of the experiment); CS, PC piglets supplemented with 40 g/t colistin sulfate; and GOD, PC piglets supplemented with 200 g/t glucose oxidase.

**Table 4:** Effect of dietary treatments on the fecal score in piglets post enterotoxigenic *Escherichia coli* (ETEC) challenge.

Treatments^1^	0 h	9 h	24 h	48 h	120 h	192 h
NC	0.60 ± 0.97	0.50 ± 0.76^b^	1.00 ± 0.82	0.88 ± 0.64^b^	0.89 ± 0.78	0.33 ± 0.50
PC	0.70 ± 1.25	2.40 ± 0.97^a^	1.78 ± 0.97	1.90 ± 0.88^a^	1.33 ± 0.87	1.22 ± 0.97
CS	0.36 ± 0.50	1.00 ± 1.18^b^	1.10 ± 0.88	0.82 ± 0.98^b^	0.64 ± 1.03	0.45 ± 0.93
GOD	0.36 ± 0.67	1.18 ± 0.98^b^	1.10 ± 0.99	1.00 ± 0.89^b^	0.91 ± 0.70	0.70 ± 1.06
*p* value	0.761	0.002	0.284	0.029	0.359	0.173

a,b Values with different superscripts within the same column differ significantly (*p* < 0.05).

1 NC, negative control (piglets were free of challenge); PC, positive control (piglets were challenged with ETEC on the 11th day of the experiment); CS, PC piglets supplemented with 40 g/t colistin sulfate; GOD, PC piglets supplemented with 200 g/t glucose oxidase.

### Serum parameters

As exhibited in Table 5, the PC group had lower (*p* < 0.05) concentrations of serum TP, glucose, and IgG with higher (*p* < 0.05) concentrations of serum ALT, IgA, and IgM than those in the NC group. Increased (*p* < 0.05) concentrations of glucose and IgG along with reduced (*p* < 0.05) concentrations of TP and IgM in serum were observed in the CS group compared with the PC group. Comparatively, there was an increase (*p* < 0.05) in serum IgG concentration together with a reduction (*p* < 0.05) of serum IgM concentration in the GOD group relative to the PC group.

**Table 5:** Effect of dietary treatments on serum parameters^1^ in piglets challenged by enterotoxigenic *Escherichia coli* (ETEC).

Treatments^2^	TP (g/L)	Albumin (g/L)	Glucose (nmol/L)	ALT (U/L)	IgG (mg/ml)	IgA (mg/ml)	IgM (mg/ml)
NC	52.46 ± 2.16^a^	29.09 ± 2.60	4.29 ± 0.49^a^	34.31 ± 3.12^b^	109.56 ± 31.54^a^ ^b^	1.83 ± 0.25^b^	27.73 ± 3.60^b^
PC	48.73 ± 3.00^b^	26.92 ± 2.37	3.46 ± 0.23^b^	42.51 ± 3.41^a^	46.20 ± 14.60^c^	2.55 ± 0.46^a^	34.42 ± 4.29^a^
CS	44.71 ± 3.78^c^	28.50 ± 2.37	4.40 ± 4.78^a^	41.40 ± 4.86^a^	117.70 ± 12.48^a^	2.52 ± 0.41^a^	24.27 ± 7.05^b^
GOD	46.40 ± 3.03^b^ ^c^	27.24 ± 2.12	3.80 ± 0.46^a^ ^b^	39.27 ± 5.08^a^ ^b^	83.18 ± 12.20^b^	2.36 ± 0.33^a^	28.25 ± 4.28^b^
*p* value	0.001	0.296	0.011	0.019	0.001	0.044	0.012

a-c Values with different superscripts within the same column differ significantly (*p* < 0.05).

1 TP, total protein; ALT, alanine aminotransferase; Ig, immunoglobulin.

2 NC, negative control (piglets were free of the challenge); PC, positive control (piglets were challenged with ETEC on the 11th day of the experiment); CS, PC piglets supplemented with 40 g/t colistin sulfate; and GOD, PC piglets supplemented with 200 g/t glucose oxidase. Samples were analyzed on the 21st day of the experiment.

### Gastrointestinal pH value

Compared with the NC group, the PC group had a reduction (*p* < 0.05) of pH value in the colon rather than in the stomach, duodenum, jejunum, ileum, cecum, and rectum (Table 6). Colonic pH value in CS group was higher (*p* < 0.05) than PC group and similar (*p* > 0.05) to NC group. Strikingly, the pH value of the stomach rather than the intestine was lower (*p* < 0.05) in the GOD group vs. the PC group but was comparable (*p* > 0.05) between the GOD group and the NC group.

**Table 6:** Effect of dietary treatments on pH value of gastrointestinal tract in piglets challenged by enterotoxigenic *Escherichia coli* (ETEC).

Treatments^1^	Stomach	Duodenum	Jejunum	Ileum	Cecum	Colon	Rectum
NC	3.91 ± 0.32^a^^,^^b^	5.11 ± 0.29	5.54 ± 0.17	6.18 ± 0.07	5.72 ± 0.09	6.10 ± 0.17^a^	6.61 ± 0.18
PC	3.97 ± 0.34^a^	5.23 ± 0.30	5.98 ± 0.17	6.28 ± 0.28	5.81 ± 0.19	5.88 ± 0.18^b^	6.38 ± 0.20
CS	4.11 ± 0.24^a^	5.17 ± 0.34	5.81 ± 0.32	6.25 ± 0.23	5.87 ± 0.21	6.09 ± 0.13^a^	6.49 ± 0.17
GOD	3.48 ± 0.36^b^	5.43 ± 0.68	5.95 ± 0.42	6.49 ± 0.24	5.79 ± 0.16	5.89 ± 0.17^b^	6.58 ± 0.07
*p* value	0.047	0.705	0.135	0.184	0.534	0.030	0.165

a,b Values with different superscripts within the same column differ significantly (*p* < 0.05).

1 NC, negative control (piglets were free of the challenge); PC, positive control (piglets were challenged with ETEC on the 11th day of the experiment); CS, PC piglets supplemented with 40 g/t colistin sulfate; GOD, PC piglets supplemented with 200 g/t glucose oxidase. Samples were analyzed on the 21st day of the experiment.

### Intestinal morphology

Duodenal and jejunal VH, as well as jejunal and ileal VCR, were lower (*p* < 0.05) in the PC group as compared with the NC group (Table 7); however, the VH of either the duodenum, jejunum, or ileum was not different (*p* > 0.05) among PC, CS, and GOD groups. Duodenal and ileal CD in both CS and GOD groups was lower (*p* < 0.05) than that in PC group but showed no difference (*p* > 0.05) from NC group. Besides, ileal VCR in the GOD group was higher (*p* < 0.05) than that in the PC group and close (*p* > 0.05) to that in the NC group.

**Table 7:** Effect of dietary treatments on intestinal morphology^1^ in piglets challenged by enterotoxigenic *Escherichia coli* (ETEC).

Treatments^2^	Duodenum			Jejunum			Ileum		
	VH (μm)	CD (μm)	VCR	VH (μm)	CD (μm)	VCR	VH (μm)	CD (μm)	VCR
NC	538.40 ± 31.35^a^	380.18 ± 40.74^a^ ^b^	1.42 ± 0.22	540.93 ± 42.20^a^	277.54 ± 19.39	1.99 ± 0.10^a^	485.98 ± 64.18	251.51 ± 11.96^b^	1.99 ± 0.24^a^
PC	485.56 ± 24.61^b^ ^c^	424.05 ± 15.49^a^	1.23 ± 0.15	445.22 ± 39.43^b^	284.98 ± 15.23	1.59 ± 0.07^b^	405.52 ± 57.17	280.38 ± 18.63^a^	1.43 ± 0.07^b^
CS	455.23 ± 27.81^c^	354.50 ± 43.60^b^	1.30 ± 0.08	441.19 ± 28.17^b^	294.37 ± 16.97	1.57 ± 0.13^b^	397.18 ± 45.17	252.77 ± 13.56^b^	1.54 ± 0.22^b^
GOD	513.53 ± 32.84^a^ ^b^	365.58 ± 26.72^b^	1.29 ± 0.12	475.79 ± 39.02^b^	285.55 ± 11.42	1.62 ± 0.12^b^	454.63 ± 10.67	246.88 ± 10.24^b^	1.85 ± 0.05^a^
*p* value	0.001	0.027	0.218	0.003	0.438	<0.001	0.052	0.007	0.001

a-c Values with different superscripts within the same column differ significantly (*p* < 0.05).

1 VH, villus height; CD, crypt depth; VCR, VH to CD ratio.

2 NC, negative control (piglets were free of the challenge); PC, positive control (piglets were challenged with ETEC on the 11th day of the experiment); CS, PC piglets supplemented with 40 g/t colistin sulfate; and GOD, PC piglets supplemented with 200 g/t glucose oxidase. Samples were analyzed on the 21st day of the experiment.

### Intestinal gene expression

**Figure 1:**
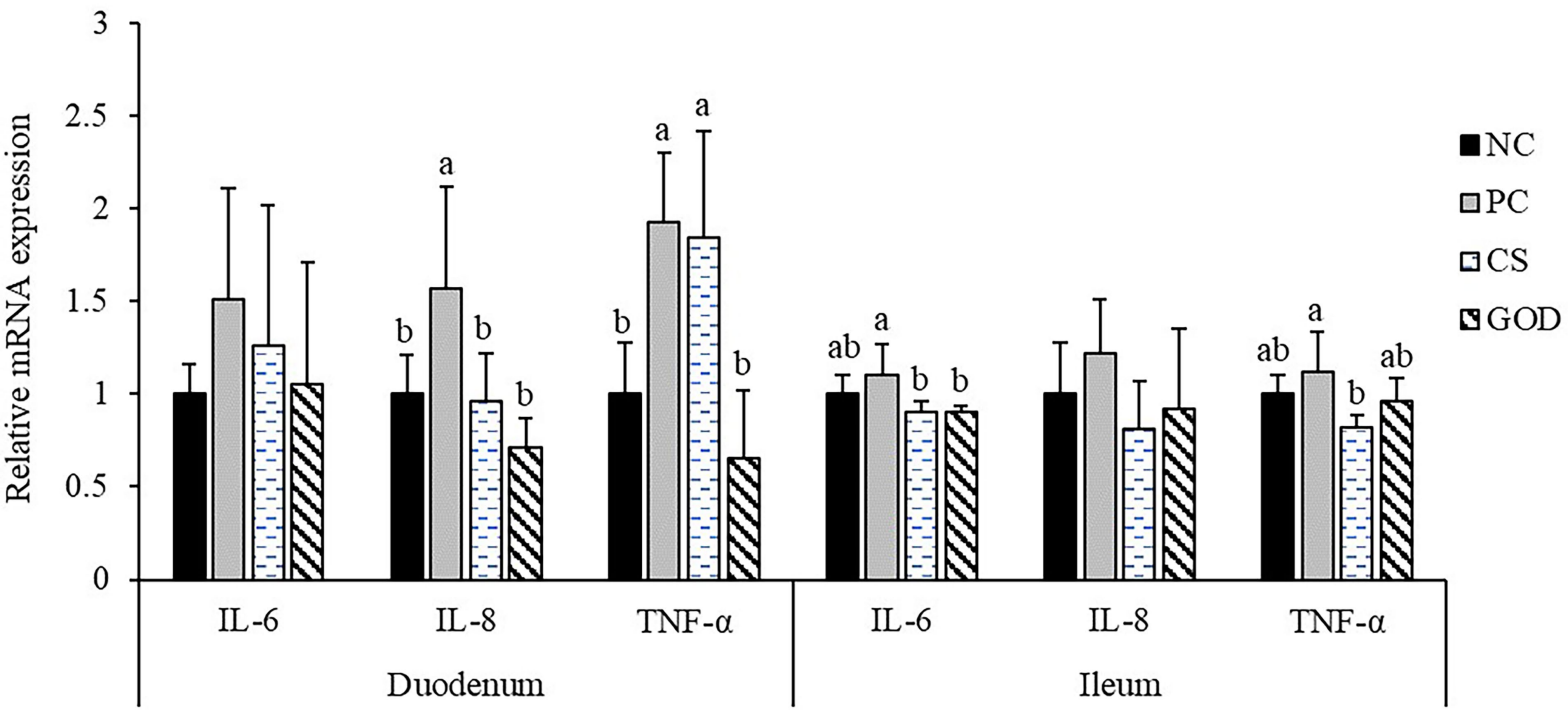
Effect of dietary treatments on the relative mRNA expression of intestinal inflammatory cytokines in piglets challenged by enterotoxigenic *Escherichia coli* (ETEC). ^a,b^Values with different superscripts differ significantly (*p* < 0.05). NC, negative control (piglets were free of the challenge); PC, positive control (piglets were challenged with ETEC on the 11th day of the experiment); CS, PC piglets supplemented with 40 g/t colistin sulfate; and GOD, PC piglets supplemented with 200 g/t glucose oxidase. Samples were analyzed on the 21st day of the experiment.

Increased (*p* < 0.05) expression of duodenal IL-8 and TNF-α was recorded in the PC group vs. the NC group (Figure 1). Both CS and GOD groups had a reduction (*p* < 0.05) in duodenal IL-8 and ileal IL-6 expression compared with the PC group. Moreover, duodenal TNF-α expression was decreased (*p* < 0.05) in the GOD group while ileal TNF-α expression was decreased (*p* < 0.05) in the CS group when compared with the PC group.

### Gut microbiota

Because the regulatory effects of antibiotics on the gut microbiota of animals have been well-known, we herein selected NC, PC, and GOD groups for gut microbiota analysis.

#### Diversity of gut microbiota

**Figure 2:**
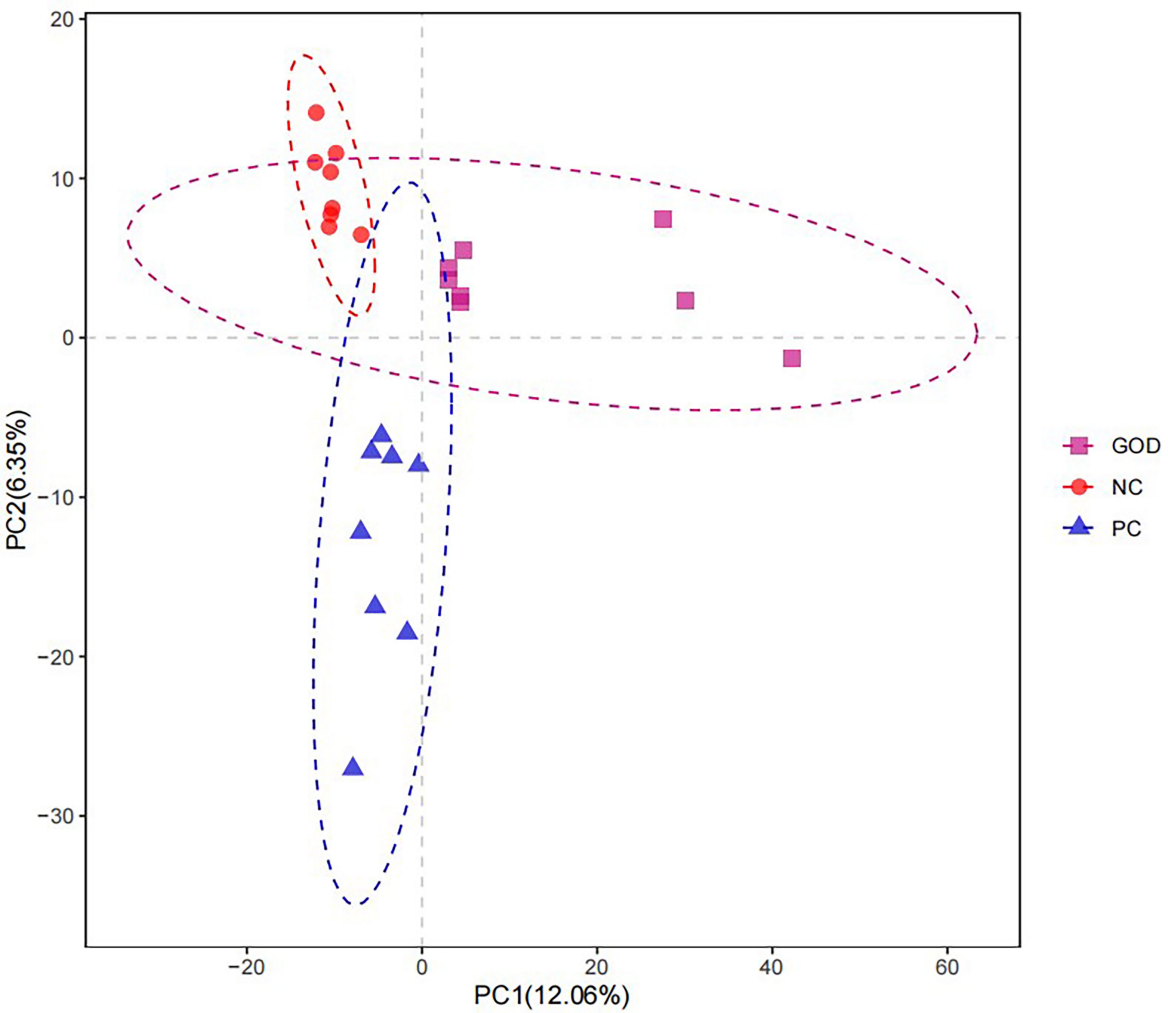
Partial least squares discriminant analysis (PLS-DA) diversity of piglet gut microbiota among groups. NC, negative control (piglets were free of the challenge); PC, positive control (piglets were challenged with enterotoxigenic *E. coli* on the 11th day of the experiment); and GOD, PC piglets supplemented with 200 g/t glucose oxidase. Samples were analyzed on the 21st day of the experiment.

No differences (*p* > 0.05) occurred in the α-diversity of piglet gut microbiota among groups (Supplementary Figure 1). Analysis of β-diversity (similarity) visualized by the PLS-DA plot displayed separation of gut microbiota among groups (Figure 2).

#### Composition of gut microbiota

**Figure 3:**
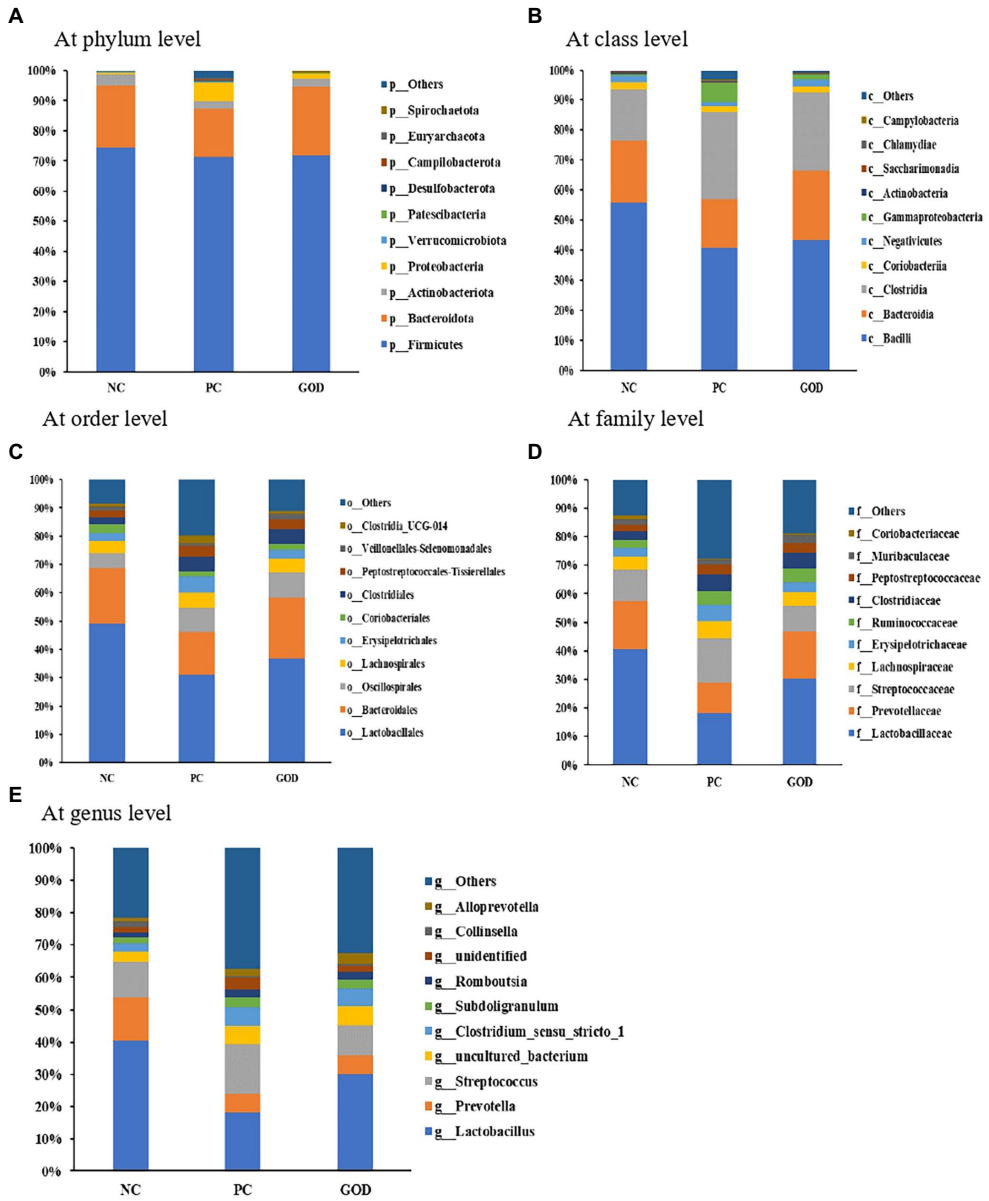
Gut microbial composition of piglets. **(A)** at phylum level; **(B)** at class level; **(C)** at order level; **(D)** at family level; **(E)** at genus level. NC, negative control (piglets were free of the challenge); PC, positive control (piglets were challenged with enterotoxigenic *E. coli* on the 11th day of the experiment); and GOD, PC piglets supplemented with 200 g/t glucose oxidase. Samples were analyzed on the 21st day of the experiment.

As shown in Figure 3, the predominant phyla in piglet gut were *Firmicutes* and *Bacteroidetes*, which accounted for greater than 90% of the whole phyla. Within *Firmicutes*, the majority belonged to the classes *Bacilli* and *Clostridia*, while the main class within *Bacteroidetes* was *Bacteroidia*. At order level, the gut microbiota was dominated by *Lactobacillales* and *Bacteroidales*. Family-level analysis manifested that the major bacteria in gut microbiota were *Lactobacteriaceae*, *Prevotellaceae, and Streptococcaceae*. At the genus level, the most abundant bacterium was *Lactobacillus* followed by *Prevotella* and *Streptococcus*.

#### Differential members in gut microbiota among groups

Plentiful bacteria at various taxonomic levels were identified as biomarkers to distinguish groups (Table 8). For example, the elevated (*p* < 0.05) proportions of several potentially harmful bacteria, such as phylum *Fusobacteriota*, class *Fusobacteriia*, orders *Fusobacteriales* and *Enterobacterales*, families *Fusobacteriaceae*, *Erysipelatoclostridiaceae*, *Enterobacteriaceae,* and *Sutterellaceae*, genera *Veillonella*, *Methanosphaera*, *Sutterella*, *Solobacterium*, *Fusobacterium,* and *Escherichia*-*Shigella*, as well as species *Veillonella_magna*, *Fusobacterium_necrophorum,* and *Escherichia_coli* in ETEC group were alleviated by GOD addition, which also attenuated the increasing trends (*p* < 0.10) of the proportions of phyla *Proteobacteria* and *Euryarchaeota*, classes *Gammaproteobacteria* and *Methanobacteria*, order *Methanobacteriales*, families *Lactobacillaceae*, *Clostridium methylpentosum*, *Eggerthellaceae,* and *Methanobacteriaceae*, together with genera *Methanobrevibacter* and *Senegalimassilia* in the ETEC group. Meanwhile, the reduced (*p* < 0.05) proportion of *Lactobacillus salivarius* along with decreasing trends (*p* < 0.10) of the proportions of *Lactobacillaceae*, *Lactobacillus*, *Lachnospiraceae_AC2044*, *Ruminococcaceae* UCG-005, *Megasphaera,* and *Megasphaera elsdenii* in GOD group were alleviated by GOD addition.

**Table 8:** Differential bacteria (%) identified from piglet gut microbiota among groups^1^.

	NC	PC	GOD	*P*-value
Phyla				
*Fusobacteriota*	0.026 ± 0.023^b^	2.379 ± 2.208^a^	0.052 ± 0.011^b^	0.024
*Proteobacteria*	0.636 ± 0.135	6.410 ± 3.439	1.694 ± 0.855	0.076
*Euryarchaeota*	0.046 ± 0.016	0.414 ± 0.250	0.061 ± 0.034	0.097
Classes				
*Clostridia*	16.866 ± 1.483^b^	29.031 ± 3.007^a^	25.799 ± 3.431^a^	0.024
*Fusobacteriia*	0.026 ± 0.023^b^	2.379 ± 2.208^a^	0.052 ± 0.011^b^	0.024
*Gammaproteobacteria*	0.632 ± 0.135	6.406 ± 3.439	1.691 ± 0.853	0.076
*Methanobacteria*	0.046 ± 0.016	0.414 ± 0.250	0.061 ± 0.034	0.097
Orders				
*Mycoplasmatales*	0.362 ± 0.250^a^	0.041 ± 0.011^b^	0.019 ± 0.012^b^	0.009
*Fusobacteriales*	0.026 ± 0.023^b^	2.379 ± 2.208^a^	0.052 ± 0.011^b^	0.024
*Clostridiales*	2.764 ± 0.450^b^	5.783 ± 1.105^a^	5.567 ± 0.790^a^	0.043
*Enterobacterales*	0.139 ± 0.054^b^	1.449 ± 0.772^a^	0.138 ± 0.037^b^	0.044
*Actinomycetales*	0.001 ± 0.001^b^	0.006 ± 0.002^a^	0.009 ± 0.003^a^	0.045
*Methanobacteriales*	0.046 ± 0.016	0.414 ± 0.250	0.061 ± 0.034	0.097
Families				
*Mycoplasmataceae*	0.362 ± 0.250^a^	0.041 ± 0.011^b^	0.019 ± 0.012^b^	0.009
*Fusobacteriaceae*	0.026 ± 0.023^b^	2.377 ± 2.206^a^	0.052 ± 0.011^b^	0.024
*Erysipelatoclostridiaceae*	0.166 ± 0.022^a^	0.233 ± 0.128^a^	0.080 ± 0.016^b^	0.024
*Clostridiaceae*	2.764 ± 0.450^b^	5.783 ± 1.105^a^	5.567 ± 0.790^a^	0.043
*Enterobacteriaceae*	0.137 ± 0.054^b^	1.448 ± 0.772^a^	0.137 ± 0.036^b^	0.044
*Actinomycetaceae*	0.001 ± 0.001^b^	0.006 ± 0.002^a^	0.009 ± 0.003^a^	0.045
*Sutterellaceae*	0.013 ± 0.006^a^	0.015 ± 0.007^a^	0.002 ± 0.001^b^	0.046
*Lactobacillaceae*	40.577 ± 4.368	18.146 ± 3.282	30.295 ± 8.332	0.055
*Clostridium methylpentosum*	0.006 ± 0.002	0.071 ± 0.026	0.019 ± 0.010	0.070
*Eggerthellaceae*	0.606 ± 0.235	0.700 ± 0.133	0.397 ± 0.133	0.085
*Methanobacteriaceae*	0.046 ± 0.016	0.414 ± 0.250	0.061 ± 0.034	0.097
Genera				
*Mycoplasma*	0.362 ± 0.250^a^	0.041 ± 0.011^b^	0.019 ± 0.012^b^	0.009
*Erysipelotrichaceae_UCG-009*	0.177 ± 0.134^a^	0.018 ± 0.008^b^	0.011 ± 0.009^b^	0.010
*Veillonella*	0.016 ± 0.011^b^	0.599 ± 0.533^a^	0.039 ± 0.008^b^	0.013
*Methanosphaera*	0.029 ± 0.011^b^	0.232 ± 0.127^a^	0.002 ± 0.002^b^	0.017
*Sutterella*	0.005 ± 0.001^a^	0.005 ± 0.003^a^	0.001 ± 0.001^b^	0.022
*Solobacterium*	0.738 ± 0.174^b^	1.282 ± 0.212^a^	0.572 ± 0.179^b^	0.022
*Fusobacterium*	0.026 ± 0.023^b^	2.377 ± 2.206^a^	0.052 ± 0.011^b^	0.024
*Escherichia/Shigella*	0.130 ± 0.054^b^	1.398 ± 0.750^a^	0.129 ± 0.035^b^	0.034
*Clostridium_sensu_stricto_1*	2.710 ± 0.442^b^	5.677 ± 1.100^a^	5.343 ± 0.784^a^	0.037
*Candidatus_Soleaferrea*	0.011 ± 0.005^b^	0.048 ± 0.013^a^	0.033 ± 0.022^a^ ^b^	0.042
*Methanobrevibacter*	0.017 ± 0.006	0.181 ± 0.128	0.059 ± 0.034	0.050
*Senegalimassilia*	0.329 ± 0.172	0.392 ± 0.118	0.136 ± 0.062	0.051
*Lactobacillus*	40.577 ± 4.368	18.146 ± 3.282	30.295 ± 8.332	0.055
*Prevotella*	13.244 ± 4.002	5.692 ± 1.997	5.805 ± 2.262	0.067
*Megasphaera*	1.097 ± 0.283	0.275 ± 0.087	2.028 ± 0.702	0.083
*Erysipelotrichaceae_UCG-003*	0.016 ± 0.005	0.006 ± 0.002	0.004 ± 0.002	0.083
*Lachnospiraceae_AC2044*	0.022 ± 0.008	0.048 ± 0.012	0.239 ± 0.143	0.088
*Ruminococcaceae UCG-005*	0.504 ± 0.095	1.505 ± 0.404	2.324 ± 1.013	0.089
Species				
*Lactobacillus_amylovorus*	23.682 ± 3.057^a^	5.522 ± 0.912^b^	8.974 ± 3.567^b^	0.008
*Metamycoplasma_sualvi*	0.359 ± 0.251^a^	0.028 ± 0.012^b^	0.019 ± 0.011^b^	0.008
*Roseburia_hominis*	0.060 ± 0.013^a^	0.049 ± 0.036^a^ ^b^	0.019 ± 0.004^b^	0.009
*Veillonella_magna_*	0.011 ± 0.008^b^	0.418 ± 0.380^a^	0.022 ± 0.003^b^	0.018
*Lactobacillus_salivarius*	0.044 ± 0.021^a^	0.006 ± 0.003^b^	0.076 ± 0.068^a^	0.019
*Fusobacterium_necrophorum*	0.024 ± 0.022^b^	2.330 ± 2.164^a^	0.051 ± 0.011^b^	0.029
*Escherichia_coli*	0.130 ± 0.054^b^	1.397 ± 0.749^a^	0.129 ± 0.035^b^	0.036
*Megasphaera_elsdenii*	1.083 ± 0.279	0.271 ± 0.085	2.011 ± 0.698	0.083

a,b Values with different superscripts differ significantly (*p* < 0.05).

1 NC, negative control (piglets were free of the challenge); PC, positive control (piglets were challenged with ETEC on the 11th day of the experiment); GOD, PC piglets supplemented with 200 g/t glucose oxidase. Samples were analyzed on the 21st day of the experiment.

It could be deduced that GOD-induced losses of *Fusobacteriota*, *Fusobacteriia*, *Fusobacteriales,* and *Fusobacteriaceae* probably originated from the reduction of *Fusobacterium,* especially the *Fusobacterium necrophorum*; the losses of *Proteobacteria*, *Gammaproteobacteria*, *Enterobacteriales,* and *Enterobacteriaceae* were primarily responsible by the decrease of *Escherichia*/*Shigella* particularly the *Escherichia coli*; the losses of *Euryarchaeota*, *Methanobacteria*, *Methanobacteriales*, and *Methanobacteriaceae* were mainly due to the reductions of *Methanosphaera* and *Methanobrevibacter*.

#### Functional prediction of gut microbiota

As exhibited in Table 9, the PC group had lower (*p* < 0.05) enrichments of the pathways of D-Glutamine and D-glutamate metabolism, fructose and mannose metabolism, purine metabolism, aminoacyl-tRNA biosynthesis, primary bile acid biosynthesis, secondary bile acid biosynthesis, pyrimidine metabolism, amino sugar, and nucleotide sugar metabolism when compared with NC group, however, the enrichments of these pathways were similar (*p* > 0.05) between GOD group and NC group. Meanwhile, supplementing GOD to PC piglets reduced (*p* < 0.05) the enrichments of the pathways of fatty acid degradation, valine, leucine, and isoleucine degradation, bacterial invasion of epithelial cells, bacterial chemotaxis, flagellar assembly, and shigellosis to levels comparable to (*p* > 0.05) those in NC piglets. Furthermore, GOD addition alleviated the decreasing trends (*p* < 0.10) of the enrichments of the pathways of starch and sucrose metabolism, taurine and hypotaurine metabolism, other glycan degradation, and sphingolipid metabolism, as well as weakened the increasing trend (*p* < 0.10) of the enrichment of lysine degradation pathway in the PC group.

**Table 9:** Comparison of the predicted pathways of piglet gut microbiota among groups^1^.

	NC	PC	GOD	*P*-value
**Nutrient metabolism-related pathways**				
D-Glutamine and D-glutamate metabolism	2.603 ± 0.042^a^	2.409 ± 0.129^b^	2.514 ± 0.210^a^ ^b^	0.009
Fatty acid degradation	0.331 ± 0.009^b^	0.371 ± 0.037^a^	0.351 ± 0.027^a^ ^b^	0.019
Fructose and mannose metabolism	1.316 ± 0.065^a^	1.105 ± 0.140^b^	1.245 ± 0.233^a^ ^b^	0.021
Linoleic acid metabolism	0.225 ± 0.027^b^	0.266 ± 0.050^a^	0.277 ± 0.032^b^	0.022
Purine metabolism	1.076 ± 0.027^a^	0.993 ± 0.053^b^	1.028 ± 0.082^a^ ^b^	0.022
Aminoacyl-tRNA biosynthesis	2.005 ± 0.042^a^	1.875 ± 0.104^b^	1.943 ± 0.178^a^ ^b^	0.029
Lysine biosynthesis	1.768 ± 0.071^a^	1.642 ± 0.076^b^	1.655 ± 0.105^b^	0.031
Primary bile acid biosynthesis	0.311 ± 0.078^a^	0.176 ± 0.063^b^	0.256 ± 0.136^a^ ^b^	0.034
Secondary bile acid biosynthesis	2.799 ± 0.701^a^	1.582 ± 0.566^b^	2.306 ± 1.225^a^ ^b^	0.034
Pyrimidine metabolism	1.458 ± 0.037^a^	1.346 ± 0.093^b^	1.412 ± 0.125^a^	0.039
Valine, leucine and isoleucine degradation	0.301 ± 0.029^b^	0.376 ± 0.063^a^	0.344 ± 0.074^a^ ^b^	0.044
Amino sugar and nucleotide sugar metabolism	1.375 ± 0.063^a^	1.226 ± 0.112^b^	1.316 ± 0.192^a^ ^b^	0.050
Starch and sucrose metabolism	1.181 ± 0.042	1.065 ± 0.108	1.104 ± 0.094	0.058
Lysine degradation	0.133 ± 0.008	0.170 ± 0.039	0.147 ± 0.031	0.069
Taurine and hypotaurine metabolism	0.889 ± 0.078	0.770 ± 0.063	0.838 ± 0.146	0.075
Other glycan degradation	0.960 ± 0.183	0.733 ± 0.248	0.974 ± 0.426	0.088
Sphingolipid metabolism	0.367 ± 0.034	0.293 ± 0.066	0.316 ± 0.135	0.099
**Bacterial pathogenicity-related pathways**				
Bacterial invasion of epithelial cells	0.002 ± 0.001^b^	0.007 ± 0.003^a^	0.001 ± 0.001^b^	0.008
Bacterial chemotaxis	0.591 ± 0.090^b^	1.107 ± 0.420^a^	0.843 ± 0.433^a^ ^b^	0.015
Flagellar assembly	0.361 ± 0.052^b^	0.678 ± 0.264^a^	0.537 ± 0.288^a^ ^b^	0.025
Shigellosis	0.001 ± 0.001^b^	0.006 ± 0.003^a^	0.001 ± 0.001^b^	0.034

a,b Values with different superscripts differ significantly (*p* < 0.05).

1 NC, negative control (piglets were free of the challenge); PC, positive control (piglets were challenged with ETEC on the 11th day of the experiment); and GOD, PC piglets supplemented with 200 g/t glucose oxidase. Samples were analyzed on the 21st day of the experiment.

## Discussion

The ETEC challenge was reported to impair growth performance in piglets (Laird et al., 2021; Pupa et al., 2022); however, it was also indicated to elicit little depression in piglet growth (Han et al., 2021a, b). In this study, ETEC-challenged piglets showed no distinct impairment in growth performance except for numerous reductions in BW and ADG. This inconsistency might result from the differences in pathogen serotype and dosage or the number of times oral ETEC administration. Previously, supplemental GOD was indicated to improve growth performance in pigs under non-infection conditions (Tang et al., 2016; Dang et al., 2021; Sureshkumar et al., 2021). However, it is unknown whether GOD could protect piglet growth against infection. Herein, we found that supplemental GOD had superiority over CS to induce an increasing trend of ADG of piglets during ETEC challenge, implying a certain protection effect of GOD on growth performance of piglets challenged by ETEC.

It is known that piglets challenged by ETEC exhibit clinical symptoms such as febrile responses and deterioration of fecal characteristics within the dozens of hours and even several days post ETEC administration (Yi et al., 2005; Lee et al., 2017). This was similar to this study in which the rectal temperature was raised at 9, 24, and 192 h post-ETEC challenge, while the fecal score was elevated at 9 and 48 h post challenge, implying time-dependent responses of body temperature and a fecal score of piglets to ETEC challenge. This agreed with previous reports (Yi et al., 2005; Lee et al., 2017) and could be responsible for the complicated relationships among ETEC colonization, shedding, and reinfection in the intestine (Yi et al., 2005). Notably, supplemental both CS and GOD reversed rectal temperature rise of piglets at 9, 24, and 192 h post challenge as well as blocked the increase in the fecal score of piglets at 9 and 48 h post challenge, which verified the role of GOD as an alternative of CS in alleviating ETEC-induced febrile responses and the potential diarrhea of piglets. These might be due to the observed mitigation of gut inflammation and inhibition of pathogen inhabitation within the intestine following GOD addition (Wu et al., 2019; Zabek et al., 2020; Dang et al., 2021).

Serum parameters are usually used to reflect the metabolism and health condition of animals. In accordance with a previous study in broilers (Liu et al., 2018), we detected reductions in serum TP and glucose levels in challenged piglets, however, both CS and GOD addition maintained the normal level of serum glucose of challenged piglets, suggesting that the increased nutrient expenditure of piglets originating from ETEC challenge could be partially alleviated by CS or GOD addition. Serum ALT activity is an indicator reflecting hepatic functional status. In this study, serum ALT activity was elevated by ETEC challenge but returned to a normal level as a result of GOD addition, demonstrating the potential of GOD to mitigate liver dysfunction in challenged piglets. This is agreed with a previous study on broilers (Wang et al., 2018). Blood immunoglobulins are important determinants of host humoral immunity (Jordan et al., 2009). It was proved that the ETEC challenge decreased serum IgG, IgM, and IgA levels in piglets (Han et al., 2021b), whereas a contrasting result was described elsewhere (Sørensen et al., 2009). In this study, there were complex responses of serum immunoglobulins in piglets to ETEC challenge, as exhibited by a reduction of IgG level concurrent with increases in IgM and IgA levels. Since IgG is the major immunoglobulin mediating humoral immune responses, the reduction of it in serum might indicate a compromise of immune defense of piglets following the ETEC challenge. To date, little is known about the effect of GOD on serum immunoglobulins levels. In this study, supplemental both CS and GOD attenuated ETEC-induced shifts of serum immunoglobulins levels especially the reduction of IgG level, indicating a role of GOD as a CS alternative in reinforcing humoral immune defense of piglets against ETEC invasion.

Increased pH value in the gastrointestine represents a negative signal for gastrointestinal health in pigs (Tugnoli et al., 2020). It was documented that the ETEC challenge increased the pH value in the stomach, small, and large intestine of piglets (Kwon et al., 2014), but a contrary finding was obtained by Slade et al. (2011). In this study, the ETEC challenge caused a minor shift in gastrointestinal pH value except for a reduction of colonic pH value, the related reason might deserve further research. Theoretically, gluconic acid produced by GOD catalysis can lower gastrointestinal pH value (Zabek et al., 2020; Zhao et al., 2022). Unexpectedly, this study showed that GOD addition reduced pH value in the stomach instead of the intestine, probably because most of the intestinal gluconic acid produced by GOD catalysis was fermented into butyric acid by certain gut microbes such as *Megasphaera elsdenii* (Tsukahara et al., 2002) that was detected to be increased in GOD group. The resulting butyric acid could be further utilized by intestinal epithelial cells and consequently caused a marginal remainder of gluconic acid in the gut. In contrast, gluconic acid produced in the stomach directly translated into a corresponding reduction of pH value, subsequently improving pepsin activity and digestion of dietary proteins (Owusu-Asiedu et al., 2003). This might partially account for the observed reduction of fecal score in challenged piglets fed with GOD.

Improvements of intestinal morphology such as increases in VH and VCR along with reduction of CD represent an expansion of villus surface area as well as acceleration in proliferation and maturity of intestinal epithelial cells, thus favoring intestinal absorption and barrier function (Mou et al., 2019). In line with previous studies (Lee et al., 2017; Liu et al., 2018), we found that ETEC challenge perturbed intestinal absorption and barrier function of piglets, as manifested by an increase in ileal CD accompanied by reductions of duodenal and jejunal VH as well as jejunal and ileal VCR. These could impair intestinal digestion and absorption leading to increased nutrient residuals in excreta with subsequent overgrowth of gut bacteria, thus coinciding with the observed increase in fecal score of challenged piglets. Several studies in pigs have validated the role of GOD in ameliorating intestinal morphology (Sun et al., 2021). Similarly, we herein noted that supplemental GOD counteracted ETEC-induced increased CD and reduced VCR of the ileum, which might be associated with the beneficial effects of butyric acid produced from GOD-catalyzed reaction on the renewal and repairing of intestinal epithelia. It is probable that the improved intestinal morphology protected intestinal absorption and barrier function in challenged piglets, thus conducing to the observed improvement of fecal characteristics induced by GOD addition.

Intestinal inflammation resulting from the ETEC challenge has been established to exert an essential role in contributing to the resultant pathologies such as febrile responses and intestinal disruption in pigs (Gao et al., 2013; Lee et al., 2017). In this study, we detected an increase in the expression of IL-8 and TNF-α of the duodenum other than the ileum in challenged piglets, suggesting a tissue-dependent effect of ETEC challenge on the expression pattern of intestinal inflammatory cytokines. Previous studies have revealed varying effects of GOD addition on intestinal cytokine expression of broilers (Qu and Liu, 2021). In this study, GOD addition counteracted ETEC*-*induced upregulations of duodenal IL-8 and TNF-α expression and also reduced ileal IL-6 expression, which validated that GOD had a capacity to alleviate intestinal inflammation in challenged piglets probably depending on the anti-inflammatory action of butyric acid produced from GOD-catalyzed reaction (Tsukahara et al., 2002; Biagi et al., 2006). Alleviated intestinal inflammation following GOD addition was favored to clarify the observed mitigatory effects of GOD on intestinal morphology impairment and febrile response in challenged piglets (Gao et al., 2013).

Gut microbiota is known as a regulator of intestinal health and growth performance of pigs (Qi et al., 2021). Similar to a previous study (Rhouma et al., 2021), this study showed that the ETEC challenge caused gut microbiota dysbiosis of piglets, as evidenced by obvious changes in β-diversity and bacterial proportions of gut microbiota, however, these changes were largely reversed by GOD addition. Among the bacteria changed by GOD addition, *Fusobacterium* spp. has a linkage with gut microbiota dysbiosis and intestinal lesion (King et al., 2020), thereinto, *Fusobacterium necrophorum* is a pathogenic bacterium having a potential to trigger intestinal necrosis of host (Ishihara et al., 2021). Expansion of *Proteobacteria* serves as an indicator of gut microbiota dysbiosis and epithelial dysfunction due to its inclusion of considerable pathogens (e.g., pathogenic *Escherichia coli*, *Salmonella,* and *Shigella*), which produce a variety of toxins leading to intestinal and systemic disorders (Litvak et al., 2017; Guo et al., 2022). Increases in intestinal *Proteobacteria*, *Gammaproteobacteria*, *Enterobacteriales*, *Enterobacteriaceae,* and *Escherichia*/*Shigella* were shown to cause intestinal inflammatory injuries (Fleckenstein et al., 2010; Litvak et al., 2017; Baldelli et al., 2021) with detriments to animal growth performance (Singh et al., 2014; Han et al., 2021a). *Methanogens*, the producers of methane, can inhibit intestinal transit and contractile activity (Pimentel et al., 2006). Several studies have verified a positive association between the increased prevalence of *Methanogens* such as *Methanosphaera* and *Methanobacteriaceae* in the gut with intestinal inflammation and inflammatory bowel diseases (Lecours et al., 2014; Yu et al., 2021). *Clostridium methylpentosum* may conduce to the gut microbiota dysbiosis-associated changes of the host during infection (Do-Nascimento et al., 2021). *Veillonella* serves as a producer of hydrogen sulfide contributing to gut microbiota dysbiosis and linked with intestinal inflammation (Said et al., 2014). *Solobacterium* acts as an opportunistic pathogen potentially leading to intestinal disorders (Alauzet et al., 2021). Accordingly, the counteractions of ETEC-induced expansions of *Fusobacteriota*, *Proteobacteria*, *Euryarchaeota,* and their affiliate members (e.g., *Fusobacterium necrophorum*, *Escherichia coli,* and *Methanosphaera*) together with *Clostridium methylpentosum*, *Veillonella* (*Veillonella magna*), and *Solobacterium* in the gut could partially interpret the observed protective effects of GOD addition against intestinal disruption of challenged piglets. Besides the above harmful bacteria, GOD also reduced or tended to reduce the families *Sutterellaceae*, *Erysipelatoclostridiaceae*, and *Eggerthellaceae*, together with genera *Sutterella* and *Senegalimassilia*. *Sutterella* spp. were suspected to participate in the pathogenesis of inflammatory bowel disease (Lavelle et al., 2015). *Erysipelatoclostridiaceae* and *Eggerthellaceae* represent opportunistic pathogens implicated in intestinal inflammation and dysfunction of the host (Shao et al., 2017; Lv et al., 2022). *Senegalimassilia* elicits a negative relationship with the utilization efficiency of nitrogen in mammals (Wang et al., 2019), while the loss of *Senegalimassilia* in the gut may mediate a prebiotic property in piglets (Dadi et al., 2020). Thus, the reductions of *Sutterellaceae* (*Sutterella*), *Erysipelatoclostr, idiaceae*, *Eggerthellaceae,* and *Senegalimassilia* in the gut could also partially account for the observed beneficial effects of GOD addition on the growth and intestinal health of challenged piglets.

In addition to decreasing the above harmful bacteria, GOD addition alleviated ETEC-induced losses in certain beneficial bacteria, such as *Lactobacillaceae*, *Lactobacillus*, *Lactobacillus salivarius*, *Megasphaera,* and *Megasphaera elsdenii*. *Lactobacillaceae* including *Lactobacillus* are typical beneficial bacteria and major sources of probiotics, exerting crucial roles in promoting intestinal health and growth of animals by restricting gut inflammation and barrier dysfunction (Jenq et al., 2012; Valeriano et al., 2017). In particular, *Lactobacillus salivarius* has the ability to protect the growth and intestinal health of pigs against ETEC infection in multiple ways (Sayan et al., 2018). *Megasphaera* spp. such as *Megasphaera_elsdenii* in pig gut can ferment gluconic acid into butyric acid (Tsukahara et al., 2002), which functions as a key nutrient and energy component for intestinal epithelial cells supporting anti-inflammation as well as repairment and renewal of intestinal tissues, thus protecting the intestine and growth of animals against challenge (Bedford and Gong, 2018). Supplemental GOD also tended to enrich *Lachnospiraceae*_AC2044 and *Ruminococcaceae*_UCG-005 that serve as important butyric acid-producing bacteria in the gut (Liao et al., 2021), probably favoring the protection of gut health and growth performance of challenged piglets. Overall, the increases in these beneficial bacteria coupled with the reductions in the above-mentioned harmful bacteria in the gut could at least partially clarify the observed conducive effects of GOD addition on growth and health parameters in challenged piglets. Similarly, limited studies in broilers revealed that GOD addition improved growth performance and intestinal health by optimizing gut microbial composition, as exhibited by the increases and decreases in certain beneficial and harmful bacteria, respectively (Wu et al., 2019; Zhao et al., 2022).

To better understand the reason for the protective effects of GOD for piglets, we focused on the comparison of the functional pathways of piglet gut microbiota among groups. The results showed that the downregulations of fructose and mannose metabolism, starch and sucrose metabolism, amino sugar and nucleotide sugar metabolism, other glycan degradation, D-Glutamine and D-glutamate metabolism, taurine and hypotaurine metabolism, aminoacyl-tRNA biosynthesis, sphingolipid metabolism, purine metabolism, and pyrimidine metabolism in the gut microbiota of challenged piglets were reversed or tended to be reversed by GOD addition, which also abolished or tended to abolish the resulting upregulations of fatty acid degradation, valine, leucine, and isoleucine degradation, along with lysine degradation. These findings disclosed that GOD addition alleviated ETEC-induced disturbances in the metabolism of carbohydrates, lipids, proteins, and nucleotides, as well as promoted macromolecular nutrients degradation and synchronously suppressed degradation of micromolecular nutrients such as fatty acids and essential amino acids especially the first limiting amino acid (lysine) for pigs, which naturally benefited energy and nutrient supply for host and subsequently supported the protective effects of GOD on piglets. Supplementing GOD to challenged piglets also weakened the downregulations of primary bile acid biosynthesis, and secondary bile acid biosynthesis, together with upregulations of bacterial chemotaxis, flagellar assembly, and bacterial invasion of epithelial cells and shigellosis. Bile acids produced by the liver are known to be secreted into intestinal lumen and metabolized by gut microbiota, playing important parts in regulating gut health such as improving lipid absorption and moderating gut inflammation (Li et al., 2022). Bacterial chemotaxis acts as a self-protective mechanism allowing the escape of bacteria from antibacterial substances produced by the host (Sourjik and Berg, 2002). Flagellar assembly is a momentous process for infection of considerable bacteria, such as ETEC, because flagella are the rotary motors driving bacterial motility (Bigot et al., 2005). It has been documented that inhibitions of bacterial chemotaxis and flagella-associated motility could be approaches to abolishing bacterial pathogenesis (Erhardt, 2016). Shigellosis is triggered by shiga-toxin-producing bacteria such as *Shigella* and ETEC, which can disrupt the intestinal structure by suppressing protein biosynthesis and promoting apoptosis of epithelial cells (Fernandez and Sansonetti, 2003; Fleckenstein et al., 2010). This was supported by the similar alteration of the pathway of bacterial invasion of epithelial cells and the observed impairment of intestinal morphology. Taken together, the normalizations of pathogenicity-related pathways (e.g., bacterial chemotaxis, flagellar assembly, bacterial invasion of epithelial cells, and shigellosis) together with the above-mentioned nutrient metabolism-related pathways in gut microbiota could be associated with the observed protective actions of the GOD addition against ETEC-induced intestinal damages, subsequently profiting improvements of growth and health performance (serum parameters and clinical symptoms) in piglets.

## Conclusion

Glucose oxidase could serve as a potential substitute for antibiotics (CS) to improve serum parameters as well as alleviate clinical symptoms and intestinal disruption in piglets challenged by ETEC. This could be at least partially responsible for its ability to reshape gut microbiota, as evidenced by the observed elevations in several beneficial bacteria and the reductions of certain harmful bacteria as well as normalizations of nutrient metabolism- and bacterial pathogenicity-related pathways. Our findings revealed the roles of gut microbiota in contributing to the protective effects of GOD against ETEC challenge in piglets, thereby providing an insight into strategies limiting *E. coli* infection in pigs. More studies with a larger sample quantity may be needed to further validate the beneficial effects of GOD on animal growth and health. Besides, the contributions of single changed bacteria in the gut to the improvement of intestinal health might be also further confirmed.

## Data availability statement

The data presented in this study are deposited in the NCBI repository, accession number PRJNA876400.

## Ethics statement

The animal study was reviewed and approved by The Animal Care and Use Committee of South China Agricultural University.

## Author contributions

WW designed the experiment and wrote the manuscript. RX conducted the animal trial and sample analysis. QC and HY assisted with sample analysis. CZ and ZD contributed to the data analysis. DF revised the manuscript. JZ supervised the research. All authors contributed to the article and approved the submitted version.

## Funding

This study was financially supported by the National Natural Science Foundation of China (No. 32102584) and the Modern Feed Industry Innovation Team Project of Guangdong Province (No. 2021KJ115).

## Conflict of interest

The authors declare that the research was conducted in the absence of any commercial or financial relationships that could be construed as a potential conflict of interest.

## Publisher’s note

All claims expressed in this article are solely those of the authors and do not necessarily represent those of their affiliated organizations, or those of the publisher, the editors and the reviewers. Any product that may be evaluated in this article, or claim that may be made by its manufacturer, is not guaranteed or endorsed by the publisher.
