# Supplementary material for: Effects of glucose oxidase on growth performance, clinical symptoms, serum parameters, and intestinal health in piglets challenged by enterotoxigenic Escherichia coli
Source: Front Microbiol. 2022 Oct 3;13:994151. doi: 10.3389/fmicb.2022.994151 (PMC9578003; doi:10.3389/fmicb.2022.994151)
Supplement: Supplementary file 1 [file Data_Sheet_1.docx]

**Supplementary Table 1.** Composition of the basal diet (air-dry basis)

| Ingredients | Content (%) |
| --- | --- |
| Corn | 64.90 |
| Soybean meal | 14.50 |
| Extruded soybean | 10.00 |
| Glucose | 2.50 |
| Fermented soybean meal | 4.00 |
| Soybean oil | 1.50 |
| Salt | 0.40 |
| Limestone | 0.98 |
| Dicalcium phosphate | 0.45 |
| L-Lysine.HCl | 0.43 |
| DL-Methionine | 0.18 |
| L-Threonine | 0.16 |
| Premix^1^ | 0.20 |
| Total | 100 |
| Nutrient levels |  |
| Digestible energy (MJ/kg) | 14.43 |
| Crude protein | 18.50 |
| Lysine | 1.34 |
| Methionine+cysteine | 0.80 |
| Threonine | 0.97 |
| Tryptophan | 0.34 |
| Calcium | 0.59 |
| Available phosphorus | 0.40 |

^1^ Supplied per kilogram of diet: vitamin A, 11500 IU; vitamin D3, 3250 IU; vitamin E, 91 mg; vitamin K_3_, 3 mg; thiamin, 8.6 mg; riboflavin, 10.9 mg; pyridoxine, 9.3 mg; cobalamin, 0.2 mg; niacin, 62.6 mg; pantothenic acid, 26.8 mg; folic acid, 1.9 mg; Cu, 9.9 mg; Zn, 142.5 mg; Fe, 242.5 mg; Mn, 68.7 mg; Se, 0.36 mg; I, 0.64 mg.

| A. | B. |
| --- | --- |
| 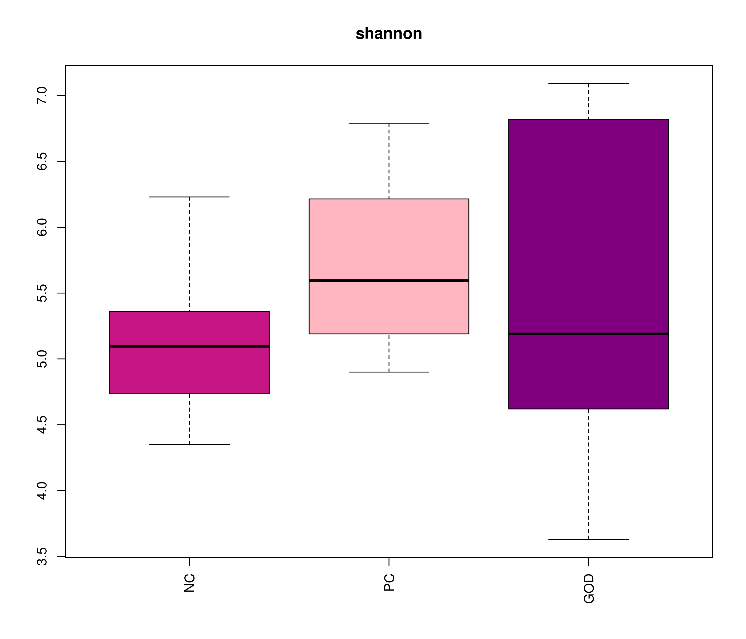 | 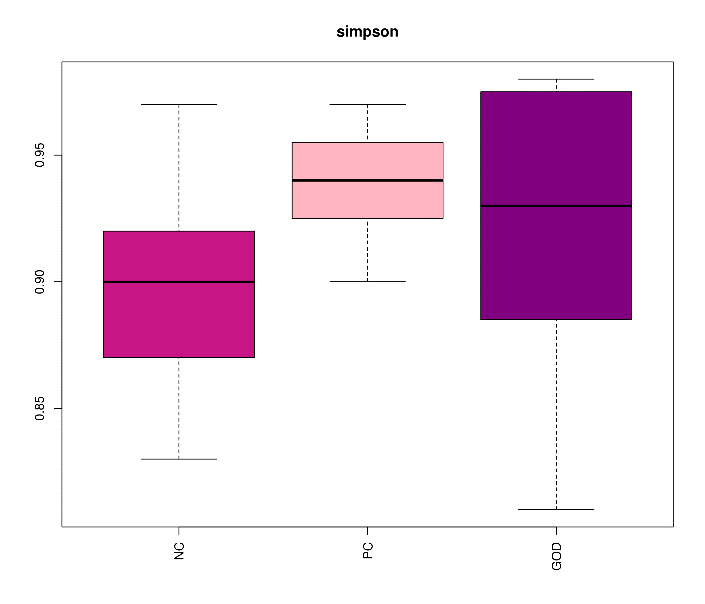 |
| C. | D. |
| 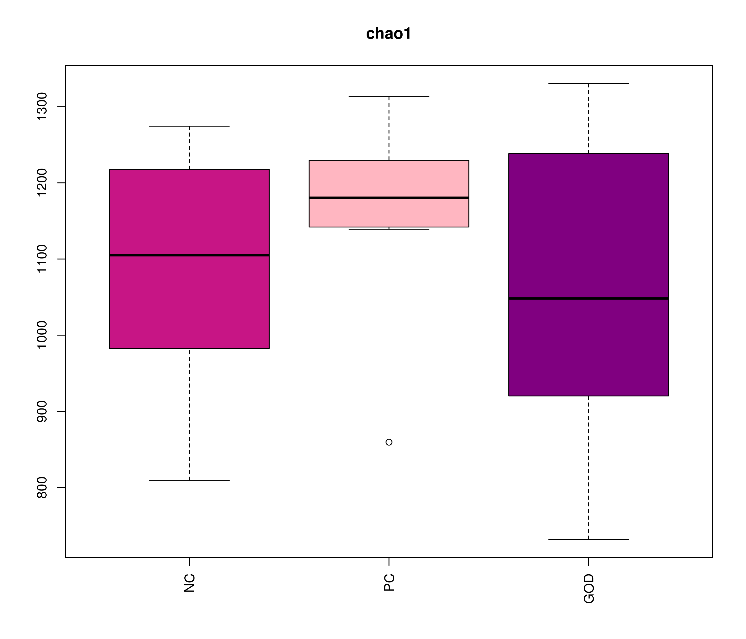 | 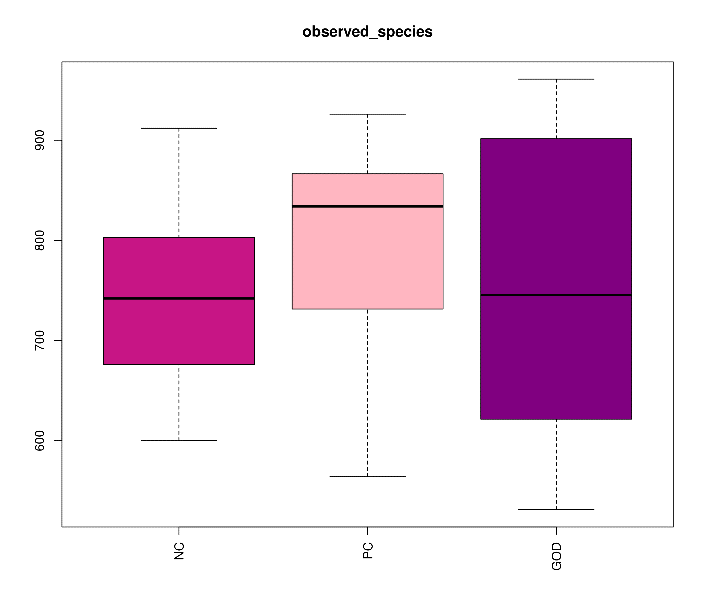 |

**Supplementary Figure 1.** Alpha diversity of piglet gut microbiota among groups. NC, negative control (piglets were free of challenge); PC, positive control (piglets were challenged with enterotoxigenic *Escherichia coli* at 11 d of the experiment); GOD, PC piglets supplemented with 200 g/t glucose oxidase. Samples were analyzed at 21 d of the experiment.
